# Pulse Decomposition Analysis of the digital arterial pulse during hemorrhage simulation

**DOI:** 10.1186/1753-4631-5-1

**Published:** 2011-01-12

**Authors:** Martin C Baruch, Darren ER Warburton, Shannon SD Bredin, Anita Cote, David W Gerdt, Charles M Adkins

**Affiliations:** 1 Empirical Technologies Corporation, PO Box 8175, 3046A Berkmar Drive, Charlottesville, Virginia, 22906, USA; 2 Cardiovascular Physiology Laboratory, 6108 Thunderbird Blvd, University of British Columbia, Vancouver, British Columbia, V6T 1Z3, Canada

## Abstract

**Background:**

Markers of temporal changes in central blood volume are required to non-invasively detect hemorrhage and the onset of hemorrhagic shock. Recent work suggests that pulse pressure may be such a marker. A new approach to tracking blood pressure, and pulse pressure specifically is presented that is based on a new form of pulse pressure wave analysis called Pulse Decomposition Analysis (PDA). The premise of the PDA model is that the peripheral arterial pressure pulse is a superposition of five individual component pressure pulses, the first of which is due to the left ventricular ejection from the heart while the remaining component pressure pulses are reflections and re-reflections that originate from only two reflection sites within the central arteries. The hypothesis examined here is that the PDA parameter T13, the timing delay between the first and third component pulses, correlates with pulse pressure.

T13 was monitored along with blood pressure, as determined by an automatic cuff and another continuous blood pressure monitor, during the course of lower body negative pressure (LBNP) sessions involving four stages, -15 mmHg, -30 mmHg, -45 mmHg, and -60 mmHg, in fifteen subjects (average age: 24.4 years, SD: 3.0 years; average height: 168.6 cm, SD: 8.0 cm; average weight: 64.0 kg, SD: 9.1 kg).

**Results:**

Statistically significant correlations between T13 and pulse pressure as well as the ability of T13 to resolve the effects of different LBNP stages were established. Experimental T13 values were compared with predictions of the PDA model. These interventions resulted in pulse pressure changes of up to 7.8 mmHg (SE = 3.49 mmHg) as determined by the automatic cuff. Corresponding changes in T13 were a shortening by -72 milliseconds (SE = 4.17 milliseconds). In contrast to the other two methodologies, T13 was able to resolve the effects of the two least negative pressure stages with significance set at p < 0.01.

**Conclusions:**

The agreement of observations and measurements provides a preliminary validation of the PDA model regarding the origin of the arterial pressure pulse reflections. The proposed physical picture of the PDA model is attractive because it identifies the contributions of distinct reflecting arterial tree components to the peripheral pressure pulse envelope. Since the importance of arterial pressure reflections to cardiovascular health is well known, the PDA pulse analysis could provide, beyond the tracking of blood pressure, an assessment tool of those reflections as well as the health of the sites that give rise to them.

## Introduction

The object of this work is the introduction of a new approach to tracking blood pressure, and pulse pressure specifically, one of the motivations being that pulse pressure appears to be a sensitive as well as specific marker for the detection of hemorrhage, [1,2] which remains one of the leading causes of death on the battlefield as well as in civilian trauma cases while also being highly preventable if intervention can be implemented [3,4]. However, detecting progressive hemorrhage requires resolution of changes on the order of a few mmHg in pulse pressures of, normally, 35-50 mmHg. Given the separate and unequal uncertainties in determining systole and diastole, using the best brachial cuff techniques, [5] such determinations are by and large out of reach even in controlled environments. The approach we present here is based on a new form of pulse pressure wave analysis, implemented through what we refer to as the Pulse Decomposition Analysis (PDA) algorithm.

The analysis of the arterial pressure pulse has been the subject of many studies, with works whose results are still relevant today dating from the 1800s and the early 1900s, [6-8] as well as a significant body of work that has been published over the past 40 or so years [9-16]. PDA presents the extension of the findings of a number of studies that have utilized ballistocardiography and invasive central artery manometers to track mechanical events such as heart contractions and pressure pulse reflections in the central arterial tree, to the arterial periphery. These studies [17-19] have confirmed the existence of two major reflection sites in the central arteries. The first reflection site is the juncture between thoracic and abdominal aorta, which is marked by a significant decrease in diameter and a change in elasticity and the second site arises from the juncture between abdominal aorta and common iliac arteries. In what follows these reflection sites are respectively referred to as the "renal" and the "iliac" reflection site.

**Figure 1:**
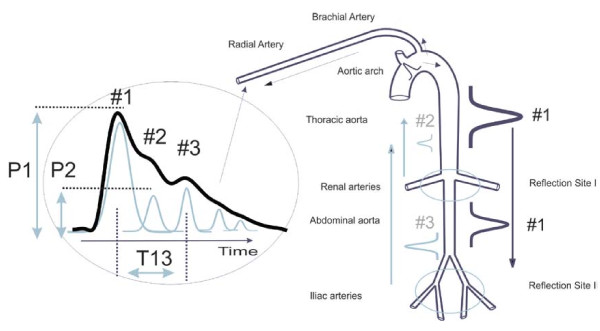
**Sketch of the aorta/arm complex arterial system and its effect on the arterial pressure pulse line shape that is observed at the radial/digital artery**. Two reflection sites, one at the height of the renal arteries, the other one in the vicinity of the iliac bifurcation, give rise to the reflected pulses (gray) that trail the primary left ventricular ejection (black).

A consequence of these reflection sites are reflected arterial pressure pulses that counter-propagate to the direction of the single arterial pressure pulse, due to left ventricular contraction, that gave rise to them. Referring to Figure 1, the "downward" travelling primary pressure pulse #1 gives rise to the "upward" travelling #2 and #3 pulses that are respectively due to the renal and the iliac reflection sites on which the #1 pulse impinged.

As these reflected #2 and #3 pressure pulses reach the aortic arch, they will enter the subclavian arteries and head into the arterial periphery of the arm, following the #1 pressure pulse that, besides traveling down the aorta, also entered the arm complex arteries. The #2 and #3 pulses will do so with certain time delays because of the "extra" traversal of the central arteries.

The #2 pulse is commonly known as the "second systolic" peak. We refer to it as the "renal reflection" and it follows the primary ejection pulse (#1) into the arterial periphery of the arm at delays of between 70-140 milliseconds. The pulse labeled #3 in Figure 1 is the much larger "iliac reflection", which follows the #1 pulse at delays of 180 to 400 milliseconds.

The described scenario succinctly explains the presence of three component pulses in the pressure pulse envelope that is observed in the arterial periphery of the arm, such as at the radial or digital arteries. In fact, there are additional component pulses. The presence of re-reflections between the central reflection sites has been previously suggested [20]. The physical picture is one where the iliac reflection pulse, in its travel up the aorta, re-reflects off the renal reflection site, and this re-re-reflection travelling downward, once again reflects off the iliac reflection site to follow the first three component pulses. With dramatically diminishing amplitude the scenario repeats for the fifth component pulse. These higher-order reflections are less relevant for quantitative analysis due to their poorer signal to noise characteristics and the fact that they are easily swallowed by the pulse envelope of the next cardiac cycle unless the heart rate is very low.

Based on these considerations the structure of the radial/digital arterial pressure pulse can be explained entirely by the interaction of the primary left ventricular ejection pressure pulse with two aortic reflection sites. We now hypothesize that it is possible to determine trends in aortic blood pressure through an analysis of the pulse envelope obtained in the arterial periphery of the arm.

The PDA model presented here analyzes the arterial pulse as observed on the lower arm by isolating, identifying and quantifying the temporal positions and amplitudes of the renal reflection pulse (#2) and the iliac reflection pulse (#3), each relative to the primary systolic pulse (#1), within the pulse shape envelope of an individual cardiac cycle. The model's predictions and experimental studies show that two pulse parameters are of particular importance. One parameter is the ratio of the amplitude of the renal reflection pulse (#2) to that of the primary systolic pulse (#1). These amplitudes, labelled P1 and P2, are indicated to the left of the arterial pressure pulse envelope. This parameter is herein referred to as the P2P1 ratio and it tracks changes in central beat-by-beat systolic pressure. The second parameter is the time difference between the arrival of the primary systolic (#1) pulse and the iliac reflection (#3) pulse. This parameter is referred to as T13, as indicated in Figure 1, and it tracks changes in arterial pulse pressure, also beat-by-beat.

It is the aim of this paper to validate the described arterial pressure pulse reflection scenario through the presentation of experimental data collected in the context of simulating central hemorrhage and its comparison with predictions of the PDA pulse propagation model. Specifically, our hypothesis is that the time delay between the primary component pulse (#1) and the iliac reflection pulse (#3), T13, correlates with pulse pressure.

We report here the results of monitoring the evolution of the PDA parameter T13 during the course of lower body negative pressure (LBNP) sessions. LBNP is an established technique used to physiologically stress the cardiovascular system. It has been used to simulate gravitational stress and hemorrhage, alter preload, and to manipulate baroreceptors [21]. LBNP was chosen for this project because it has been shown to be very effective at modulating pulse pressure, thereby providing a means to validate the equivalent PDA arterial pulse parameter, T13.

## Patients and Methods

After IRB approval, tests of the CareTaker™ system, which is the hardware implementation of the PDA model that is described in more detail below, were performed at the Cardiovascular Physiology Laboratory of the University of British Columbia on fifteen healthy volunteers (average age: 24.4 years, SD: 3.0 years; average height: 168.6 cm, SD: 8.0 cm; average weight: 64.0 kg, SD: 9.1 kg) whose lower bodies, from the height of the iliac crest downwards, were subjected to increasingly negative pressures. A number of studies have demonstrated that it is possible to simulate significant internal hemorrhage using LBNP. Negative pressures of 10-20 mmHg correspond to 400 to 550 ml of central blood loss, 20-40 mmHg correspond to 500 to 1000 ml, and negative pressures in excess of -40 mmHg correspond to blood losses exceeding 1000 ml [22].

The subjects were subjected to four stages of negative pressure, -15 mmHg, -30 mmHg, -45 mmHg, and -60 mmHg, each stage lasting typically about 12 minutes. The blood pressure was monitored with an automatic cuff (BP TRU Automated Non-Invasive Blood Pressure Monitor (model BPM-100), VSM MedTech Devices Inc.) set to record blood pressures every three minutes, resulting in typically four readings per LBNP setting as well as an Ohmeda 2300 Finapres, and a pulse oximeter (Ohmeda Biox 3740 Pulse Oximeter, BOC Health Care) monitored oxygen saturation. The CareTaker system collected arterial pulse shapes beat-by-beat via a finger cuff attached to the central phalange of the middle digit. Four subjects became presyncopal and could not complete the -60 mmHg LBNP stage.

### A. CareTaker Device

The hardware platform that provides the arterial pulse signal for the PDA algorithm's analysis is the CareTaker™ device (Empirical Technologies Corporation, Charlottesville, Virginia). It is a physiological sensing system whose three basic physical components are a sensing pad such as a finger cuff that couples to an arterial pressure point, a pressure line that pneumatically telemeters the pulsations, and a custom-designed piezo-electric pressure sensor that converts the pressure pulsations, using transimpedance amplification, into a voltage signal that can be measured, digitized, transmitted and recorded. The coupling to the artery is accomplished using palpation coupling, such as at the radial artery, or approximate hydrostatic coupling, such as at the digital arteries. The completely self-contained device wirelessly transmits its signal representing the arterial pulse to a PC computer using the Bluetooth protocol. The device is not occlusive as it operates at a coupling pressure of about 40 mmHg. Another important characteristic of the device is that the signal it provides, sampled at 512 Hz, is the *time derivative *of the arterial pulse signal. The derivative provides significant signal to noise advantage and lowers the resolution requirements for digital acquisition of the signal because the derivative eliminates signal offsets. Because of the short time constants associated with its implementation, it also offers very short recovery times after signal disruptions. That is, in the absence of offsets due to the differentiation, the signal is always clamped to the signal base line, which in turn allows for increased amplification. Consequently the full digitization range of the analog to digital converter (A/D) can be used for the signal amplitude, as opposed to signal amplitude plus offsets.

### A The PDA model

The existence of two distinct central pressure pulse reflection sites make it is possible to propose a simple model of the arterial paths that the primary pulse and its reflections traverse and to compare its predictions with observations regarding the relative arrival times of the different components pulses. The model's equations predict the time of arrival of each individual component pulse, subject to the total distance that the pulse has travelled and the *pressure-dependent *pulse propagation velocity in each arterial segment. The different relevant arterial paths are denoted by x_n_, where x_1 _refers to the arm arterial path while x_2_, x_3 _refer to the thoracic and abdominal aorta, respectively. t_n _refers to the time of arrival of the nth component pulse at the radial/digital arterial peripheral site. While in the case of the #1 pulse its arrival time, t_1_, is determined only by its travel along the arm complex arteries (x_1 _path), the arrival times for the #2 and #3 pulses take into account their initial travel as the primary ejection pressure pulse, travelling at systolic pressure, as well as, after impacting a reflection site, their subsequent return as a reflected pulse at pressures that are now significantly lower. As an example, the "second systolic" (#2) pulse traverses the thoracic aorta at systolic pressure, traverses it again as an R2 reflection after re-direction at the renal arteries reflection site (indicated as R2 of pulse pressure plus diastolic pressure) and then enters the arm arteries where it loses another percentage of its amplitude due to the R1 reflection coefficient that incorporates artery segment transitions, such as the aortic/subclavian junction.

The pressure dependence of the pulse propagation velocity is implemented using the Moens-Korteweg [23] equation, specifically ν = √((hE e^ζ P^)/(2ρα)) relating pressure and velocity. Its definitions are as follows: ν_x_(P) is the velocity of the xth arterial pulse path as a function of the pressure P_x,m _indicated, where × is again the index of the path section and m is a running term index. In the definition of ν E is the Young's modulus, α is the artery's diameter, h is the arterial wall thickness, ρ is the fluid density, ξ is the arterial compliance and P is the pressure and PP is the pulse pressure. The Young's modulus and the arterial extensibility ξ are different for the different arterial segments.

(1) $$t_{1}=\frac{x_{1}}{v_{x_{1}}(P_{11})},$$

(2) $$t_{2}=\frac{x_{2}}{v_{x_{2}}(P_{21})}+\frac{x_{2}}{v_{x_{2}}(P_{22})}+\frac{x_{1}}{v_{x_{3}}(P_{23})}$$

(3) $$t_{3}=\frac{x_{2}}{v_{x_{2}}(P_{31})}+\frac{x_{3}}{v_{x_{3}}(P_{32})}+\frac{x_{3}}{v_{x_{3}}(P_{33})}+\frac{x_{2}}{v_{x_{2}}(P_{34})}+\frac{x_{1}}{v_{x_{1}}(P_{35})}$$

(4) $$P_{11}=P_{syst}-R_{1}\;P_{Pulse}$$

(5) $$P_{21}=P_{syst}$$

(6) $$P_{22}=P_{diast}+R_{2}\;P_{Pulse}$$

(7) $$P_{23}=P_{diast}+R_{2}(1-R_{1})P_{Pulse}$$

(8) $$P_{31}=P_{syst}$$

(9) $$P_{32}=P_{syst}-R_{2}\;P_{Pulse}$$

(10) $$P_{33}=P_{diast}+R_{3}(1-R_{2})P_{Pulse}$$

(11) $$P_{34}=P_{diast}+R_{3}(1-R_{2})(1-R_{2})P_{Pulse}$$

(12) $$P_{35}=P_{diast}+R_{3}(1-R_{2})(1-R_{2})(1-R1)P_{Pulse}$$

Another critical feature of the model is that R2, the renal reflection coefficient, is dependent on systolic pressure. The motivation for this is based on the following consideration. As discussed, the renal reflection (P2 pulse) originates at the junction between thoracic and abdominal aorta, a junction that is characterized by a significant change in arterial diameter. Since the thoracic aorta is the softest artery in the body, as evidence by the fact that it exhibits the lowest pulse pressure propagation velocities (4-5 m/s) and much more extensible than the abdominal aorta, increasing peak pressure, or systole, will enlarge the diameter mismatch, giving rise to a more pronounced renal reflection pulse amplitude while falling systole will produce the opposite effect, an effect observed in manipulative experiments performed by Latham [17]. The critical insight then is that the amplitude of the renal reflection will increase relative to the amplitude of the primary systolic (P1 pulse) peak because, while both component pulses travel the arteries of the arm complex, and are therefore both subject to the pulse narrowing and heightening due to the taper and wall composition changes of the peripheral arteries, only the renal reflection will have sampled the pressure-induced aortic impedance mismatch changes. This establishes the motivation for taking the ratio of the amplitudes of the #2 and the #1 pulse, which is P2P1.

**Figure 2:**
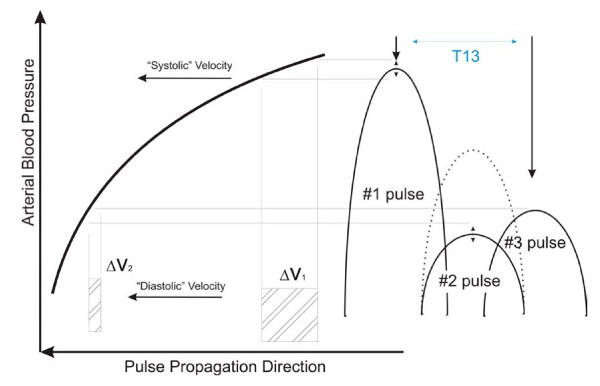
**Relative amplitudes and velocity/pressure relationships of the component pulses**. #2's amplitude and propagation velocity can change significantly due to the pressure dependence of R2, the renal reflection coefficient.

A similarly physical argument can be made for the difference in arrival times of the primary pulse (#1) and the iliac reflection (#3), or T13. The difference in the arrival times of the primary arterial pulse, that is the left ventricular ejection, and the iliac reflection pulse is determined by the differential velocities with which both pulses propagated along their arterial paths. In the case of the iliac reflection the path length is longer than that of the primary pulse by almost twice the length of the torso. More importantly, both pulses travel at different velocities because their pressure amplitudes are different. Specifically, the iliac reflection pulse amplitude, which is determined by the reflection coefficient of the iliac reflection site, is on the order of 40% of pulse pressure. This point is graphically made in Figure 2. Both pulses therefore load the arterial wall differently during their arterial travel, as a result of which their propagation velocities are different. The second insight is that, because the pressure/velocity response curve is non-linear, a result known since the 1960s based on Anliker's work, [24] both pulses accelerate and decelerate at different rates as the pressure rises and falls. The primary pulse experiences the highest changes in velocity as a function of changes in blood pressure because it is subject to the steepest section of the pressure/velocity response curve, while the iliac pulse, "running" at much lower pressure, changes velocity much more gradually. Changes in the time of arrival therefore then reflect changes in the differential arterial pressure that the two pulses experience. While this differential pressure is not exactly pulse pressure, that is the difference between the full pulse arterial pulse height and the diastolic pressure floor, it represents about 60%-70% of it, assuming the previously stated iliac reflection coefficient.

**Figure 3:**
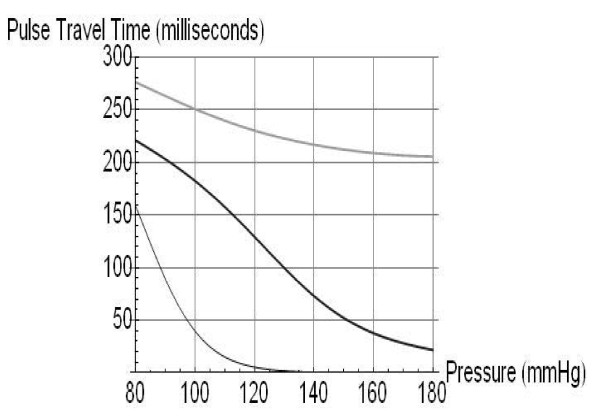
**Arrival times, as predicted by PDA model, of the three component pulses, from bottom up, #1 (left ventricular ejection), #2 (renal reflection), and #3 (iliac reflection)**.

Figure 2 presents a graphic display of the relative amplitudes of the left ventricular ejection (#1) and the trailing reflection pulses and their resulting relative positions on the pulse propagation velocity curve, which is highly pressure dependent. As a result the arrival times of the different pulses are highly pressure dependent, a point that is clarified by Figure 3, which presents the pulse travel times, from bottom to top, respectively, of the primary ejection pulse (#1), the renal reflection (#2), and the iliac reflection (#3). The iliac pulse's arrival time shortens only slightly with increasing pressure because its amplitude remains close to the diastolic pressure regime. The renal reflection peak's arrival time (middle) experiences significant non-linearity because the reflection coefficient, R2, is highly pressure dependent. The left ventricular ejection (#1, bottom curve) has the highest amplitude and samples the steepest section of the pressure/velocity curve and is therefore most pressure dependent. Using Young's moduli obtained from the literature and letting the model fit R2 as well as the velocities of the primary arterial path ways it is then possible to compare experimental data with model predictions.

Figure 3 displays the fact that human arterial pathways, for the average height population we have studied, are generally very short relative the distances the arterial pulse traverses within a cardiac cycle. Typical arterial pulse propagation velocities range, for healthy and unstressed arteries, from 4 - 9 m/s. This fact influences particularly the arrival time of the #1 pulse profoundly. In the lower pressure range, which is the pressure regime that was examined here, the #1 pulse pulls away from the #2 and #3 reflection pulses, as evidenced by the fact that its arrival time shortens significantly faster with increasing pressure than the arrival times of #2 and #3. Consequently, in this pressure range, T13 would be expected to widen with increasing pressure and shorten with decreasing pressure. Figure 3 therefore provides a quantitative basis for why T13 is hypothesized to be directly dependent on blood pressure changes in the blood pressure regime that was examined here.

As the pressure continues to increase, however, the arrival time of the primary #1 pulse saturates as it runs out of arterial runway. Consequently further increases in arterial pulse propagation velocity do not result in a further shortening of the arrival time. Meanwhile the #3 pulse continues to accelerate with increasing pressure, narrowing the T13 time delay in this high-pressure regime. The details of the pressure-dependent evolution of the arrival time curves are critically dependent on the choice of different velocity profiles for the different arterial sections, a point that is discussed later.

### B. Pulse Decomposition Algorithm (PDA)

The algorithm that is based on the pulse analysis model just presented encompasses the following components: 1. a peak finder that identifies heartbeats in the derivative data stream, 2. a differentiator that produces the second derivative of the detected heart beat which is then used to find the inversions corresponding to the locations of the component pulses, 3. a digital integrator, implemented as a Bessel filter, that generates the integrated pulse wave form from the differentiated raw signal stream, and from which relative component pulse amplitudes are determined and 4. a low-pass filter that allows identification of the primary systolic peak. Furthermore the frequency content of the data stream is continuously analyzed in order to calculate signal to noise (S/N) figures of merit that determine whether signal fidelity is sufficiently high to permit peak detection and analysis.

The detection efficiency of the heart beats was typically on the order of 92%, as evidenced by visual inspection of inter-beat spectra which readily reveal missed beats. Detection was typically poorest at the highest negative pressure (-60 mmHg) because of significantly diminished pulse amplitude.

Once the temporal locations of the reflection component pulses and the systolic peak are identified, the T13 interval, the time delay between systolic (P1) and iliac peak (P3), is calculated. The P2P1 ratio is calculated using the amplitudes of the P2 peak and the systolic peak, in the integrated pulse spectrum. Detection efficiency of the component pulses was on the order of 90%. Detection again was poorest at the highest negative pressure because of diminished pulse amplitude.

### C. Statistical Analysis

We present regression coefficients between LBNP levels and pulse pressure responses of the three measurement systems. In order to compare relative sensitivities of the three systems to changes in pulse pressure we present results of different repeated measures ANOVA analyses, which were performed using the Minitab statistical software (Release 14, Minitab Ltd.). Data are presented as means ± SE unless specified otherwise.

## Results

### A. Comparison of Pulse Pressure Changes

**Figure 4:**
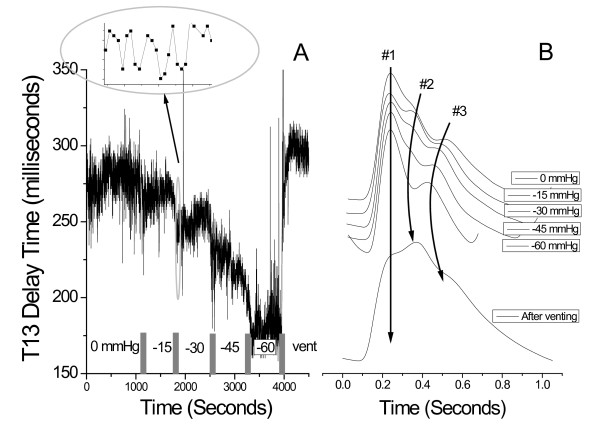
**Evolution of T13 parameter (graph A) and corresponding arterial pressure pulse line shapes (10-pulse averages) for subject #5, 31 y. f, over the course of an entire LBNP session**. The narrowing of the arterial pulse is indicated by the downward (with time) pointing arrows that identify the changing temporal position of component pulses #2 and #3 relative to the primary systolic pulse #1. In this subject the heart rate changed significantly. Note the different rate of change in the T13 interval and the heart rate. Of interest also is the massive rebound that is observed in the #2 component pulse amplitude after venting, which subsided within minutes.

In Figure 4 we present an example of the evolution of arterial pressure pulse line shape changes for the 6 stages of an hour-long LBNP session (right-hand graph B) as well as the T13 trace for the entire session (left graph A). The subject in this case was a 31 y. female. The time evolution of the presented pulse line shapes is downward, starting at the top at atmospheric pressure, and ending with a pulse line shape obtained after the LBNP chamber was vented from -60 mmHg back to atmospheric pressure. Each pulse line shape represents a 10-pulse average.

The dynamic range of the iliac and renal peak positions is indicated by the downward sweeping arrows, while the position of the primary systolic peak (#1) is indicated by the vertical solid arrow. The narrowing of the time interval between iliac and systolic component pulses with decreasing negative pressure is clearly visible. Furthermore, while the heart rate also changed, as indicated by the shortening inter-beat interval, it is clear that the *rates of *change for T13 and heart rate are different, i.e. the inter-beat interval narrowed faster than T13. A further point of interest is the shape of the arterial pressure pulse after venting, which in all subjects caused a significant rise in systolic blood pressure, as determined using the conventional blood pressure monitors. The pressure pulse line shape in question has the typical pulse shape associated with a positive augmentation index, which is defined as (height of #2 pulse - height of #1 pulse)/maximum overall amplitude[25]. A positive augmentation index is usually taken to be indicative of arterial aging, which, given the subject's age, is unlikely to be the case. This subject's pulse shape returned, along with normalizing systole, within minutes to the original line shape (top trace in Figure 4B).

**Figure 5:**
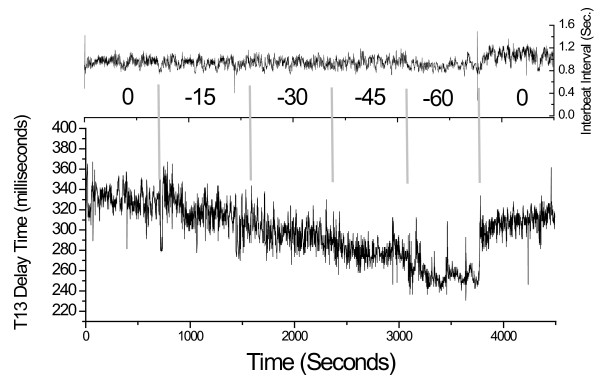
**Temporal evolution of the inter-beat interval and T13 over the course of the LBNP session of subject #9, 24 y. m., In this subject the narrowing of T13 was observed without any change in heart rate until venting**.

While the results displayed in Figure 4 exhibited a significant change in heart rate along with the change in T13, this was not a general observation. Figure 5 presents the results regarding inter-beat interval and T13 for subject #9, a 24 y. male, who did not exhibit any appreciable change in heart rate until venting. The narrowing of T13 with decreasing negative pressure, however, matched those of all subjects.

**Figure 6:**
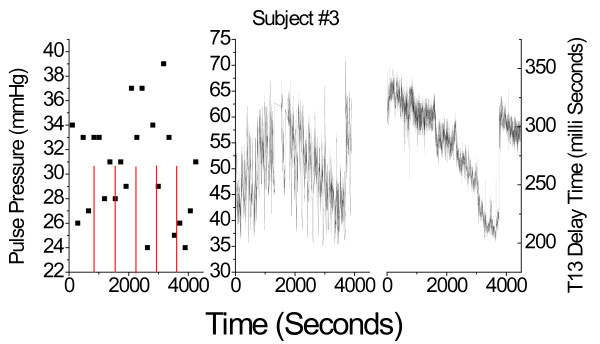
**Comparison of the individual results for cuff-based pulse pressure (left graph), Finapres-based pulse pressure (center), and PDA-based T13 measurements, for subject #3**. The right panels present the simultaneously obtained T13 delay times between the primary left-ventricular ejection pulse and the iliac reflection pulse recorded on the subject's middle member of the middle digit.

Figure 6 displays a representative side-by-side comparison of pulse pressures obtained with the automatic cuff (left graph) and the Finapres (center graph), as well as the evolution of the T13 parameter over the course of the LBNP session of subject #3, (right graph). The general absence of a discernible trend in the readings of the cuff with progressing hypovolemia was typical for all data runs.

**Figure 7:**
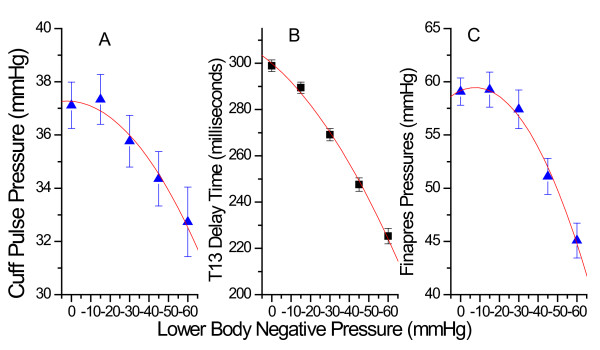
**Overall results for pulse pressure obtained with the automatic cuff (graph A), the PDA pulse pressure-equivalent parameter T13 (B) and the Finapres (C)**.

Figure 7 presents comparative overall results for pulse pressures and T13 as a function of progressive decreasing negative pressure. Specifically, Figure 7A presents the overall pulse pressure results of the automatic pressure cuff while Figure 7B presents the overall results for T13. Figure 7C presents overall pulse pressure results for the Finapres.

The ability of the four measurement methods to resolve the effects of the different LBNP stages at a statistically significant level varied. While the PDA T13 parameter was able to resolve each of the four LBNP stages relative to atmospheric pressure, neither the Finapres nor the cuff were able to resolve the stages with the two least negative pressures (-15 & - 30 mmHg), corresponding to the smallest changes in pulse pressure, with significance set at p ≤ 0.01. Heart rate, as a detection modality for resolving the two least negative pressure LBNP stages, almost reached statistical significance, performing significantly better than the Finapres or the cuff. Table 1 presents the results of the ANOVA analysis.

**Table 1:** ANOVA: PDA, Finapres, Cuff versus LBNP (-15 & -30 mmHg)

Methodology	Significance
PDA - T13	0.00001
Finapres pulse pressure	0.636
Cuff pulse pressure	0.214
Heart rate (Finapres)	0.02

**Figure 8:**
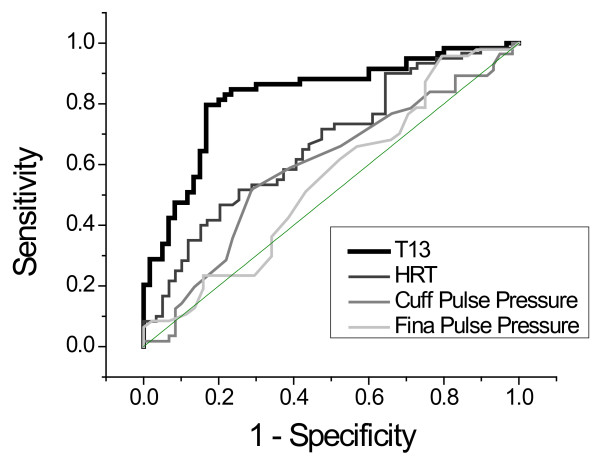
**ROC analysis of the comparative intra-subject sensitivity/specificity of T13, heart rate, cuff pulse pressure, and Finapres pulse pressure to resolving the difference of -15 mmHg versus -30 mmHg LBNP relative to atmospheric pressure**. Respectively the areas under the curve are 0.83, 0.66, 0.59, and 0.55.

A receiver operating characteristic (ROC) analysis of the intra-subject ability of the four methodologies to resolve LBNP-induced differences of -15 mmHg and atmospheric pressure, and -30 mmHg and atmospheric pressure revealed similar differences. In Figure 8 we present the results.

**Figure 9:**
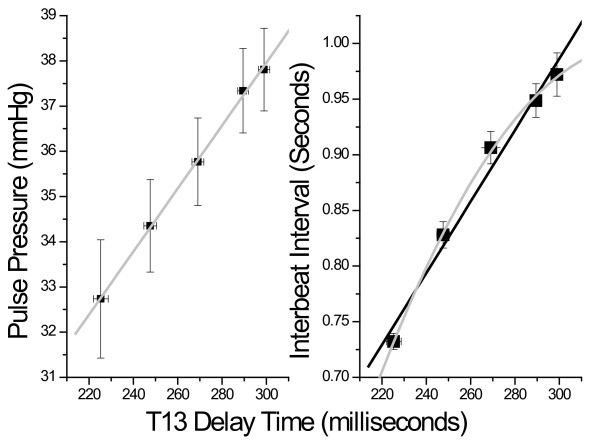
**Functional comparison of T13 with pulse pressure obtained from cuff (left), and heart rate (right)**.

An important question is whether T13 is indeed a pulse pressure equivalent or whether it simply tracks the changes in heart rate referred to in Figure 4. Figure 9 presents a comparison of pulse pressure, as measured with the cuff, as a function of T13 and heart rate, as measured with the Finapres, as a function of T13. While T13 correlates linearly with the pulse pressure determined using the cuff (0.19 × T13 (milliseconds) + 2.58, R^2 ^= 0.98, p < 0.0001) a second order model is required to obtain a correlation with heart rate (-1.51x T13 (milliseconds) 0.015 -2.24526E-5 T13^2 ^(milliseconds)).

### B. Comparison with model predictions

**Figure 10:**
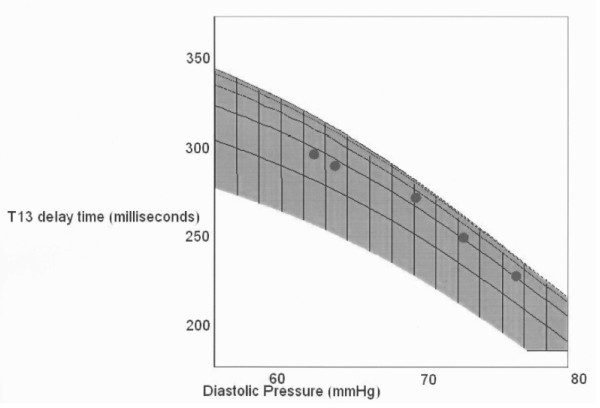
**Overlap of PDA T13 prediction and LBNP study data as a function of diastolic pressure change. The line around which the data are grouped corresponds to systole = 120 mmHg**. Adjacent lines correspond to ±5 mmHg.

In Figure 10 we present an overlay of the experimental results and the model's predictions. The experimental data, all averages from 15 subjects, are the T13 values obtained from each LBNP stage as well as the corresponding pulse pressure values as determined with the Finapres. Since systole did not change appreciably for any of the subjects, we use the average value of 120 mmHg throughout. Consequently, as observed experimentally, changes in pulse pressure are driven entirely by changes in diastole. The most important aspect of the agreement between the model and data, as presented in Figure 10, are the arterial parameter assumptions that are required to achieve it. The single dominant factor that determines the response of T13 to pressure changes is the pressure/arterial pulse velocity response of the different arterial sections that the systolic pulse and its two central reflections traverse. Furthermore the range of relative pressure/velocity response curves that is possible, given the constraints of the experimental data, is very narrow. Clearly the model at this stage uses a significant simplification of the arterial path sections and the response curves presented represent averages over these pathways. While more details will be introduced in future versions of the model the aim here is to demonstrate that the basic physical picture hypothesized by the PDA model matches observations.

While the starting values of the pressure/velocity curves for the arm complex arteries as well as the thoracic/abdominal aorta were based on published arterial pulse propagation velocities, [26] the results of the LBNP experiments provide an opportunity to deduce the relative dynamic response characteristics of the different sections, which are not readily available as they have not been the subject of research interests in a long time. In order to obtain the fit shown in Figure 10, a different dynamic behavior of the arm complex relative to that of the central arteries had to be modeled. Specifically, while the arm complex arteries required a distinct exponential response characteristic, the simulated central arteries' response, in the blood pressure range under consideration, was essentially linear. And it is this difference in dynamic response that enables the model to generate T13 curves whose slopes match those observed. In contrast, changes in starting values only shifted the family of curves in parallel up or down in but did not change the relative slopes of the curves.

## Discussion

Before discussing the implications of what appear to be statistically significant correlative results it is important to consider the hypothesis of whether they could be due to an unrelated experimental artefact, specifically the increasing abdominal compression with the increasing LBNP pressures that has been reported [27]. Two arguments can be made to refute this concern. If the increasing abdominal compression were to have progressively given rise to a new reflection site between renal and iliac reflection sites, a new reflected component pulse would have arisen between the #2 and #3 component pulses, progressively increasing with each LBNP-induced abdominal compression stage. Such additional central reflection sites have been observed by Kriz [20] in the context of aortic aneurysms. We have observed the resulting additional component pulses of such aortic aneurysms in the arterial periphery, a subject of future publication. However, as part of this work, in none of the LBNP stages and in none of the subjects studied were such additional component pulses observed.

A second argument is that the same response in T13 is observed in the case of actual hemorrhage, induced through blood donation of 1 pint. With two subjects only so far, the continuous decrease of T13 was observed as the blood donation progressed. Moreover, the decrease observed in the blood donation experiment matched well the average decrease that was observed in the first stage (-15 mmHg) of the LBNP results reported here, in line with the estimation of central blood loss for that stage [22]. A blood donation study with 50 subjects to verify these preliminary results is about to commence.

The results presented therefore support the hypothesis that the time delay between the primary component pulse (#1) and the iliac reflection pulse (#3), T13, correlates with pulse pressure and provide a first milestone in the validation of the PDA model. A number of conclusions follow.

Any reflection sites in the arm complex arteries proximal to the radial/digital arteries will not affect the pulse line shape that is observed there because any pulse reflections due to such reflection sites will travel *away *from them and back toward the central arteries. Their re-entry into the arm complex arteries could only be accomplished as re-reflections, with dramatically reduced amplitudes that would be masked by the primary renal and iliac reflections.

The hypothesis that the shape of the peripheral pressure pulse is predominantly determined by reflections in the aorta may seem surprising and will no doubt be gradually accepted. On the one hand maneuvers that are known to modify the thoracic pressure profile, such as valsalva that selectively modulates the renal reflection site and therefore the #2 component pulse, [17] provide a ready method to demonstrate the critical importance of the central reflection sites. Alternatively manipulative experiments in the arterial periphery could be suggested to support or challenge the hypothesized physical picture. One possibility is partial occlusion of a femoral artery, with pulse monitoring distally on, for example, the pedal artery. Particularly since in the legs the #2 and #3 component pulses are diminished because they are re-reflections, it should be possible to observe the additional component pulse that would originate from the partial occlusion site, travel to the iliac reflection site and reflect there toward the distal monitoring site. Movement of the occlusion site along the leg arteries should change the timing of the additional component pulse relative to the pulse envelope.

A relevant physiological phenomenon is pressure pulse amplification of the arterial periphery that is attributed to the taper of the arterial walls as well as their changing wall composition relative to the core arteries. The important realization here is that, while the arterial pressure pulse is temporally compressed and increased in amplitude, these changes to the pulse envelope are, in the absence of arterial dilation or constriction, static. This is the basis of using the validated transfer function approach [26] that uses pulse shapes obtained in the arterial periphery to predict central artery pulse shapes and blood pressures. It is the central artery dynamics that determine the peripheral pulse in the transfer function model. In the PDA model, which offers a concrete physical model instead of a generalized Fourier-based inverse filter, it is also the central artery dynamics that dominate the relationships between the components. Significant arterial dilation and constriction does modify the component pulse relations, and this is an object of current study.

The head plays *a much diminished role *in regard to pressure pulse reflections that are observed at the arterial periphery of the arm. Arterial pressure pulse reflections that return via the carotid arteries will, upon entering the aortic arch and traveling along the descending thoracic, re-reflect off the reflection site in the vicinity of the renal arteries. Assuming a reflection coefficient on the order of 17%, the amplitude of such a re-reflection will be on the order of 3% of the primary peak amplitude, and consequently be masked by the much larger pressure primary pulse reflections #2 and #3.

**Figure 11:**
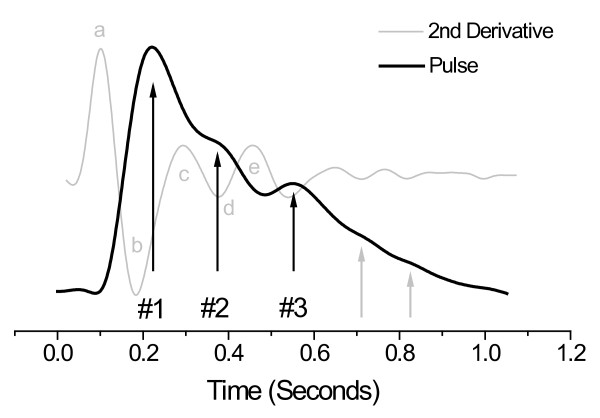
**Arterial pressure pulse line shape (black trace, 10-pulse average), and its second derivative (gray trace), of a 21 y. male athlete collected at the middle phalange of the middle finger**. The pulse line shape displays distinct pulsatile features labeled #1, #2, #3. While the #1 is the direct pass due to left ventricular ejection, the rest of the pulse envelope is due to arterial reflections. Indicative of distinct arterial pulse reflection sites is the fact that the reflected wave exhibits pulsatile components (#2, #3, and beyond) that feature comparable temporal extents as the primary ejection-related feature #1. The inversions of the second derivative trace are labeled according to the convention introduced by Takada *et. al*.

The PDA model ties together recent related observations by others. The fact that the ratio of the amplitudes of the #2 (P2) and #1 (P1) pulses correlates with systolic pressure is not surprising in light of the results obtained by Takazawa et. al., [28] Takada et. al., [29] and Imanaga et al. [30] In Figure 11, which displays the 10-pulse average of the arterial digital pressure pulse of a 21 y. male athlete, we also present the second derivative of the arterial pulse. Takazawa et. al. labeled the different inversions, "waves", of this second derivative trace as indicated in the figure. The results of several studies suggest that the ratio d/a correlates with blood pressure, along with many other physiological parameters [28,29]. Comparative inspection of the two traces establishes that the waves "a" and "d" are temporally in very similar positions to P1 and P2, respectively. A parameter introduced by Bartolotto [25] that incorporates *very similar definitions *of P1 and P2 as the PDA model was also found to correlate with systolic pressure. This parameter is the augmentation index of the photoplethysmograph, PTG (AUGI), and it is defined as (P2-P1)/MA, where P2 and P1 are the amplitudes of the primary and second systolic peaks in the photoplethysmograph, respectively, and MA is the maximum envelope amplitude. These correlative results support the PDA model, which supplies the physical explanation both for the origin of the component pulses as well as why the correlation of the relative amplitudes of these component pulses with systolic pressure exists.

As with the PDA's P2P1 parameter, others have suggested measures that utilize the same time interval corresponding to T13 and have somewhat comparable physical interpretations. Millasseau [31] labels the time delay PPT in the digital volume pulse and suggests that it corresponds to the transit time of pressure waves from the root of the subclavian artery to the "apparent" site of reflection "in the lower body" and back to the subclavian artery. The reason for choosing the subclavian artery as a starting and ending point is however unclear, since the pressure wave does not originate there. If, on the other hand, the subclavian artery were to give rise to the #3 pressure pulse as a reflection site, the amplitude of the iliac pulse would be dramatically lower than what is observed (20-40% of the primary peak) at the radial or digital artery. Succinctly put, the pulse would have travelled from the left ventricle to the subclavian artery, reflected there at some reflection site, then to travel to the iliac reflection site. It would return from there as a re-reflection pulse with commensurately much reduced amplitude, an unlikely scenario.

A significant benefit of measuring T13 over pulse pressure directly is its higher resolution and sensitivity. The results indicate the equivalence of a change of about 200 milliseconds in T13 to a variation of about 8 mmHg in pulse pressure *over the entire range *of a simulated central blood loss in excess of 1 liter for this cohort of fit and relatively young subjects. The results therefore indicate that the PDA technology is capable of resolving small changes in pulse pressure, a feat that sphygmomanometers are not well suited for. Given the suggestion by others that pulse pressure can be considered as a surrogate for stroke volume and therefore as a means to track loss of blood volume in trauma patients, [1] the accurate monitoring of pulse pressure could be a vital component in predicting hemorrhagic shock.

The potential benefits of utilizing T13 in detecting small changes in pulse pressure, coupled with the small size of the wireless CareTaker hardware, which weighs on the order of 5 oz, and the fact that it tracks blood pressure at low coupling pressures, makes the system attractive for the monitoring of patients at risk for internal hemorrhage. A benefit of such field-based monitoring is that internal hemorrhage could be detected well before hemodynamic collapse, making timely intervention feasible.

Currently studies are underway to further validate the PDA model by simultaneously correlating intra-aortic blood pressure with the peripherally measured PDA parameters T13 and P2P1.

## Conclusions

We have presented a new physical model of the propagation of the arterial pressure pulse and its reflections as well as a comparison of the predictions of the model with experimentally obtained pulse parameters and conventionally obtained blood pressures. The agreement of observations and measurements provides a preliminary validation of the model which in turn could provide a renewed impetus in the study of the human arterial pressure pulse. The model is based on few, physical, assumptions because it proposes that the structure of the pulse is due to it is readily identifiably arterial pulse reflection sites. As a result it is also readily testable.

## Competing interests

Drs. Baruch, Gerdt, and Adkins are the founders of and are fully employed by Empirical Technologies Corporation and have developed both the CareTaker hardware as well as the PDA formalism. Since the CareTaker is a commercial device they stand to gain financially if the PDA formalism is validated and accepted.

Drs. Warburton and Bredin and Ms. Cote are with the Cardiovascular Physiology Laboratory, University of British Columbia, Vancouver, British Columbia. Neither they nor the Laboratory has received funds from ETC nor do they stand to gain financially if the CareTaker technology proves successful.

## Authors' contributions

MCB data analysis, model development, and drafting of manuscript. DERW data collection, editing and revision of manuscript. SSDB data collection; AC: data collection. DWG model refinement, editing and revision of manuscript; CMA model refinement, editing and revision of manuscript. All authors read and approved the final draft.
